# Gene Mutations Associated With Chronic Lymphocytic Leukemia (CLL) Among Saudi CLL Patients and Treatment Outcomes: A Single-Center Experience

**DOI:** 10.7759/cureus.59044

**Published:** 2024-04-26

**Authors:** Mohammed A Alsayari, Giamal Edein M Gmati, Aamir Omair, Abdullah Alhobabi, Faisal T Alanazi, Mohammed A Almutairi, Al Waleed K Al Faifi

**Affiliations:** 1 College of Medicine, King Saud Bin Abdulaziz University for Health Sciences College of Medicine, Riyadh, SAU; 2 Oncology, King Abdullah Medical City, Riyadh, SAU; 3 Research, King Abdulaziz Medical City Riyadh, Riyadh, SAU; 4 Medical Education/Research, King Saud Bin Abdulaziz University for Health Sciences, King Abdullah International Medical Research Center, Ministry of National Guard Health Affairs, Riyadh, SAU

**Keywords:** 17q deletion, 13q deletion, trisomy 12, cytogenetic aberrations, chronic lymphocytic leukemia (cll)

## Abstract

Background

Chronic lymphocytic leukemia (CLL) starts in white blood cells in the peripheral blood (stages 0 and 1). In CLL, leukemia cells often build up slowly. Many gene mutations are associated with CLL, such as trisomy 12, 13q14 deletion, and 17q deletion. Due to the lack of patients' disease characteristics, gene mutations, and treatment outcomes data among Saudi patients, this study aimed to identify the relation between the gene mutations of CLL and the treatment in King Abdulaziz Medical City (KAMC), Riyadh.

Methods

This cross-sectional study used data from the BESTCare hospital information system. The study included all patients diagnosed with CLL and confirmed by flow cytometry in KAMC, Riyadh, between January 2010 and October 2020. The data included demographic information, mutation type or chromosome, present comorbidity, and type of treatment.

Results

The study included 100 CLL patients. According to different types of clusters of differentiation (CD), CD5 was positive in 84 (84%) patients, and 88 (88%) patients were positive for CD19. Cytogenetic remarkers were tested, revealing that 21 (21%) patients with trisomy 12 and 20 (20%) were positive for 13q14 deletion. Observation of patients’ disease status based on the cytogenetic remarkers showed that out of 15 patients with trisomy, 12 (80%) had not progressed and were stable and alive. Out of 20 patients with 13q14 deletion, 16 (80%) were alive and 13 (65%) patients were stable.

Conclusion

CLL patients in KAMC, Riyadh, displayed trisomy 12, which is characterized by the worst prognosis of disease status, as the most frequently detected cytogenetic aberration followed by 13q deletion. However, most patients were stable and alive.

## Introduction

Chronic lymphocytic leukemia (CLL) is the most common type of leukemia in elderly patients. It is characterized mainly by the accumulation and clonal proliferation of mature B-cells in the blood, bone marrow, and spleen and the disability to undergo apoptosis [1,2]. The etiology of CLL is still idiopathic. Contrarily to the other types of leukemia, CLL is unaffected by exposure to radiation or any cytotoxic agents [1].

CLL is considered the most common type of leukemia in the Western hemisphere, and the median age of diagnosis is approximately 70 years. In the United States of America and Canada, the 5-year survival rate for people aged 20 years and older with CLL is around 84%, and in Europe, it is 68% for men and 74% for women [1,3-5]. In the Kingdom of Saudi Arabia, CLL is one of the most common types of leukemia. Also, over 15 years (1999-2013), the Saudi Cancer Registry (SCR) registered 750 cases of patients with CLL [6]. The distribution of CLL cases based on the regions in this registry showed that the highest cases were in the Central region, followed by the Southern, Western, and Eastern regions [6].

The detection of genetic abnormalities in CLL patients has considerably improved over the last few years. Several tests have proved their reliability in the detection of CLL gene mutations. One is fluorescence in situ hybridization (FISH), which detects a specific part of the gene in the DNA [2,5,7,8]. DNA sequencing detects P53 gene mutation, which stops tumor cell growth, by determining the nucleic acid sequence and the order of nucleotides in DNA [2,5,7]. Another test used in CLL diagnosis is bone marrow aspirate and trephine biopsy, which is also done in some cases before commencing treatment in CLL patients [2,5,7]. Some gene mutations, such as the p53 gene, are associated with a higher risk of some types of cancer, including CLL [9-11]. Other types of mutations that may occur secondary to a P53 mutation are deletion of chromosome 13 (del(13q)), deletion of chromosome 17p (del(17p)), and trisomy of chromosome 12 (+12) [9-11].

Management of CLL is mainly to 'watch and wait' for asymptomatic patients. Treatment in CLL patients is indicated for patients with bulky lymphadenopathy, anemia when hemoglobin is less than 10 gm/dl, thrombocytopenia when platelets are less than 100 x109/L, and the lymphocytes doubling time is less than six months [1,2,7,12,13]. Based on five prognostic variables (age, serum β2-microglobulin, clinical stage, IGHV mutational status, and TP53 status), the International Prognostic Index for Chronic Lymphocytic Leukemia (CLL-IPI) categorized patients with CLL into four risk groups. These groups are as follows: low-risk patients with an overall survival (OS) rate of approximately 93.2% at five years, intermediate-risk patients with an OS rate of about 79.3%, high-risk patients with an OS rate of around 63.3%, and very high-risk patients with an OS rate of approximately 23.3% over five years [12,13]. Therapeutic options for CLL include a combination of venetoclax with obinutuzumab, chemotherapy, immunotherapy such as monoclonal antibodies, and targeted therapy such as proton kinase inhibitors. Combinations of chemotherapy and immunotherapy, for instance, the fludarabine, cyclophosphamide, and rituximab (FCR) protocol, which is considered to be the first standard of care for young CLL patients, can be used depending on the stage of the disease, patient's age, and performance status [1,2,7,12,13]. At relapse, the second line of therapy, which usually includes drugs that have not been used before, can be considered [12,13]. An allogeneic stem cell transplant (SCT) is another therapeutic option for young fit CLL patients with multiple relapses or failed treatment on several lines of therapy, especially patients with higher risk or poor cytogenetic markers [12,13].

Due to the lack of patients' demographics, disease characteristics, gene mutations, and treatment outcomes data among Saudi patients, we conducted this study to determine the various cytogenetic abnormalities of CLL patients in King Abdulaziz Medical City (KAMC), Riyadh, the association of the outcome of treatment, and to confirm if there is a relationship between cytogenetic abnormalities and disease status.

## Materials and methods

This retrospective study was conducted in the adult hematology division at King Abdulaziz Medical City in Riyadh (KAMC-RD). A complete list of patients' medical record numbers (MRNs) was obtained primarily from flow cytometry laboratory records and crosschecked against tumor board discussion sheets. KAMC-RD was inaugurated in 1982 and is considered one of the most comprehensive healthcare medical cities in Saudi Arabia. It provides all levels of care, from public health and primary healthcare to the most advanced tertiary services.

The study subjects included all patients diagnosed with CLL and confirmed by flow cytometry (FC) in KAMC between January 2010 and October 2020. However, all patients with no cytogenetic or molecular report were excluded at the time of diagnosis. With a 10% margin of error, confidence level of 95%, and expected outcome percentage of 50%, the required sample size was estimated to be 100 patients.

The study received Institutional Review Board (IRB) approval from King Abdullah International Medical Research Center with approval number H-01-R005. Participant confidentiality was observed throughout the study using serial numbers for each subject. Patient data was obtained from the BESTCare system and medical notes in conjunction with a tumor board recommendation sheet for the cases diagnosed with CLL before 2015. The data included demographic information (age, gender), chromosomal anomalies (trisomy 12, deletion of chromosome 13q14 (del(13q14)) or chromosome 17p (del(17p)), present comorbidity, and type of treatment (anti-CD20 antibodies, chemotherapy, and phosphoinositide 3-kinase inhibitor).

Data were collected in a Microsoft Excel sheet (Microsoft Corporation, Redmond, WA, US), exported, and analyzed in Statistical Package for the Social Sciences (SPSS; IBM Corp., Armonk, NY, US). Categorical variables were presented as frequencies and percentages, and outcomes were compared between the different groups using the chi-square test. A p-value of <0.05 was considered to show a statistically significant association.

## Results

A total of 100 patients were included; their mean age was 70.7 + 11.9 years and 67 (67%) of them were males (Table 1). There were 69 (69%) patients who had no lymphadenopathy while 17 (17%) had cervical lymphadenopathy. Regarding hepatomegaly and splenomegaly, 71 (71%) of the patients had palpable liver while 61 (61%) had palpable spleen. At the time of diagnosis, 23 (23%) of patients admitted with Rai stage 0, 40 (40%) with Rai stage I, 19 (19%) with Rai stage II, 10 (10%) with Rai stage III, and 8 (8%) with Rai stage IV.

**Table 1:** Characteristics of chronic lymphocytic leukemia patients (n=100)

Variables		n	%
Age	(mean + sd)	70.7 + 11.9	
Gender	Male	67	67%
	Female	33	33%
Lymphadenopathy	No lymphadenopathy	69	69%
	Cervical	17	17%
	Axillary	6	6%
	Cervical & axillary	8	8%
Hepatomegaly	Not palpable	29	29%
	Palpable	71	71%
Splenomegaly	Not palpable	39	39%
	Palpable	61	61%
Rai stage	0	23	23%
	I	40	40%
	II	19	19%
	III	10	10%
	IV	8	8%

**Figure 1:**
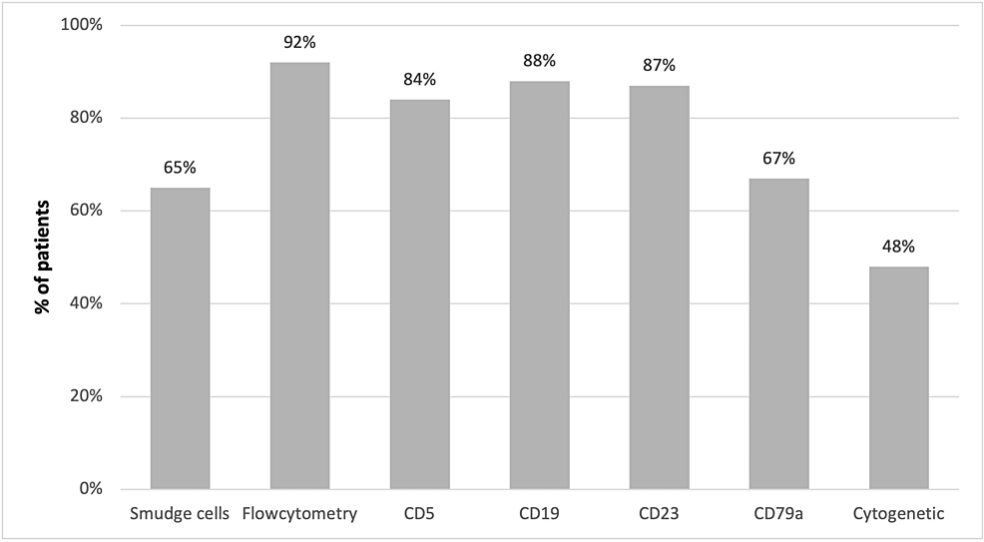
Presence of Smudge cells, molecular abnormalities in flow cytometry, and immunophenotype of chronic lymphocytic leukemia patients (N=100)

**Figure 2:**
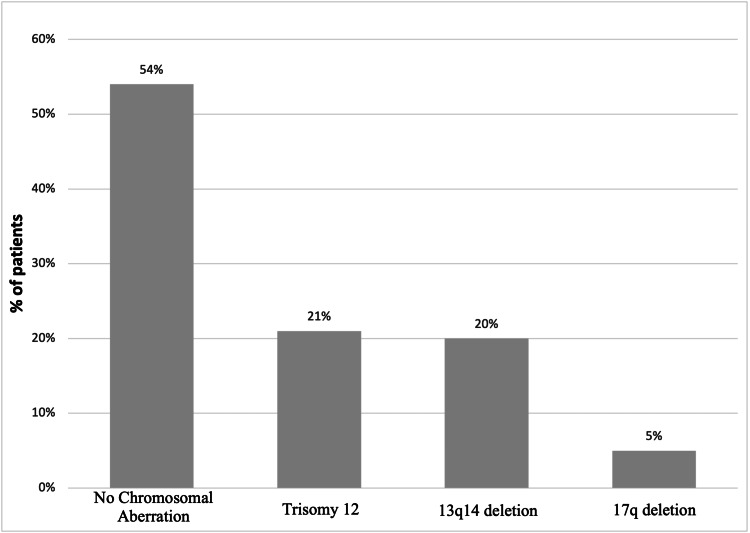
Chromosomal aberration of chronic lymphocytic leukemia patients (N=100)

Figure 1 shows that 65% of the patients tested positive for smudge cells. Additionally, 84% of the patients exhibited a positive presence of cluster of differentiation 5 (CD5), 88% tested positive for CD19, 87% were found to be positive for CD23, and 67% tested positive for CD79a. In Figure 2, there were 54 (54%) patients with no cytogenetic remarkers, 21 (21%) were positive for trisomy 12, there were 20 (20%) positive for 13q14 deletion, and 5 (5%) were positive for 17q deletion.

Table 2 shows various treatment options used for CLL patients, including 'watch and wait', which is the most common initial management plan; some were given single medication treatment, a combination of medications, treatment, or stem cell transplant depending on the patient's status. According to Table 3, which shows the disease status of CLL patients, 75 (75%) of the patients were stable, 66 (88%) of stable patients were alive, and 9 (12%) were dead. However, 25 (25%) CLL patients suffered from disease progression, 14 (56%) were alive, and 11 (44%) patients died. Table 3 also shows an association between the disease and patient status (p=0.001).

**Table 2:** Treatment options used for chronic lymphocytic leukemia patients

	Treatment 1	Treatment 2	Treatment 3	Treatment 4
Watch and Wait	73	10	7	3
Ibrutinib	5	5	12	3
Fludarabine	1	1	0	0
Rituximab	3	6	4	3
Chlorambucil	2	8	4	5
Imatinib	0	1	1	0
Intravenous immunoglobulin (IVIG)	1	1	3	0
Obinutuzumab	0	0	0	1
Stem cells transplant	1	0	0	0
Ibrutinib & Rituximab	2	1	0	0
Fludarabine & Rituximab	2	4	0	0
Cyclophosphamide & Rituximab	0	1	0	0
Rituximab & Chlorambucil	2	1	1	2
Fludarabine. Cyclophosphamide. Rituximab (FCR)	7	3	1	0
Rituximab & Bendamustine	0	5	1	1

**Table 3:** Relation between disease status and outcome of chronic lymphocytic leukemia patients The table shows an association between the disease and patient status of p=0.001, which shows significance. The p-value is significant if < 0.05.

		Total	Alive/Dead		p-value
			Alive	Dead	
Disease status	Progression	25	14	11	0.001
			56%	44%	
	Stable	75	66	9	
			88%	12%	
Total		100	80	20	

Table 4 illustrates the association between cytogenetic markers and disease status, with a p-value of 0.09. Among a total of 54 patients lacking cytogenetic markers, nine patients (17%) experienced disease progression while 45 patients (83%) remained stable. In the case of 21 patients exhibiting trisomy 12, six patients (29%) showed disease progression while 15 patients (71%) remained stable. Among the 20 patients with a 13q14 deletion, seven patients (35%) progressed while 13 patients (65%) remained stable. Lastly, among the five patients with a 17q deletion, three patients (60%) experienced progression while two patients (40%) remained stable. In Table 5, among a total of 54 patients who lacked cytogenetic markers, nine individuals (17%) passed away while 45 (83%) remained alive. In the case of 21 patients who presented trisomy 12, six patients (29%) died while 15 patients (71%) survived. Among the 20 patients with a 13q14 deletion, four patients (20%) did not survive while 16 patients (80%) were alive. Lastly, among the five patients with a 17q deletion, one patient (20%) passed away and four patients (80%) remained alive.

**Table 4:** Relation between cytogenetic aberration and disease status in patients with chronic lymphocytic leukemia (N=100) Since the p-value is significant if < 0.05, this table shows no significance.

		Total	Disease status		p-value
			Progression	Stable	
Remarker 1	No chromosomal aberration	54	9	45	0.09
			17%	83%	
	Trisomy 12	21	6	15	
			29%	71%	
	13q14 deletion	20	7	13	
			35%	65%	
	17q deletion	5	3	2	
			60%	40%	

**Table 5:** Relation between cytogenetic remarkers and alive/dead status in patients with chronic lymphocytic leukemia (N=100) Since the p-value is significant if < 0.05, this table shows no significance.

		Alive/Dead				p-value
		Alive		Dead		
		n	%	n	%	0.72
Cytogenetic Remarkers	No Remarker	45	83%	9	17%	
	Trisomy 12	15	71%	6	29%	
	13q14 deletion	16	80%	4	20%	
	17q deletion	4	80%	1	20%	

## Discussion

The most frequent chromosomal aberration detected in chronic lymphocytic leukemia (CLL) patients included in the study was trisomy 12, followed by 13q deletion, then 17q deletion. In contrast, a local study on CLL patients in King Faisal Specialist Hospital (KFSH) showed 13q deletion as the most aberration detected, followed by trisomy 12 and 17q deletion, respectively [14]. Furthermore, a study conducted in Germany to determine genomic aberrations in CLL patients found that 13q deletion was the most detected change, followed by trisomy 12, then 17q deletion [15]. Also, another study conducted in China on distinct age-related clinical features and risk assessment in Chinese with CLL supports the same result [16].

Regarding the cytogenetic changes and treatment outcomes, this study illustrates that most patients with 17q deletion had the worst prognosis, followed by 13q deletion, while trisomy 12 had a slightly better prognosis. However, a study done to identify cytogenetics in chronic lymphocytic leukemia suggested that patients with trisomy 12 had the worst prognosis while 13q deletion patients had a better outcome [17].

This study concluded that most CLL patients were on Rai stage I at diagnosis. As a result, the 'watch and wait' option was the preferred initial treatment option used, followed by the fludarabine, cyclophosphamide, and rituximab (FCR) regimen. Ibrutinib was the treatment of choice for relapsed CLL patients. Another study published about current treatment options in CLL showed that 'watch and wait' was the preferred treatment for patients first presented and diagnosed with Rai stages 0 and I without active disease [18]. In addition, ibrutinib was the drug of choice used for relapsed patients with refractory CLL [18]. Another study supported the findings of this study using FCR as a first line of treatment for CLL patients requiring therapy [19].

The study's limitations can be summarized in several points. First, the sample size of the study was small. Also, KAMC was the only center where patients were included, so the results cannot be representative. Another limitation is that some data are lacking because the primary source of study data, the BESTCare system, which is the hospital information system (HIS), was launched in 2015. So, data before this date were written in doctor’s notes in the hospital archive, and some data were missing.

## Conclusions

There was no considerable association between cytogenetic abnormalities and CLL patients' treatment outcomes. It is recommended that further studies with a larger sample size from multiple health centers should be conducted to improve the treatment outcomes in CLL patients with poor cytogenetic markers.
